# IFN-Alpha Receptor-1 Upregulation in PBMC from HCV Naïve Patients Carrying CC Genotype. Possible Role of IFN-Lambda

**DOI:** 10.1371/journal.pone.0093434

**Published:** 2014-04-01

**Authors:** Eleonora Lalle, Licia Bordi, Claudia Caglioti, Anna Rosa Garbuglia, Concetta Castilletti, Chiara Taibi, Francesca Cristofari, Maria Rosaria Capobianchi

**Affiliations:** 1 Laboratory of Virology, National Institute for Infectious Diseases “Lazzaro Spallanzani,” Rome, Italy; 2 Clinical Department, National Institute for Infectious Diseases “Lazzaro Spallanzani,” Rome, Italy; Fondazione IRCCS Policlinico San Matteo, Italy

## Abstract

**Background and Aims:**

IL-28B gene polymorphisms predict better therapeutic response and spontaneous clearance of HCV. Moreover, higher expression of IFN-lambda has been reported in patients with the rs12979860 CC favourable genotype. The study aim was to establish possible relationships between IL-28B rs12979860 genotypes and expression of IFN-alpha receptor-1 (IFNAR-1) in naïve HCV patients, and to explore the possible role of IFN-lambda.

**Methods:**

IFNAR-1 mRNA levels were measured in PBMC from naïve patients with chronic hepatitis C with different IL-28 genotypes. The ability of IFN-lambda to up-regulate the expression of IFNAR-1 was established in PBMC from healthy donors carrying different IL-28B genotypes.

**Results:**

Lower IFNAR-1 mRNA levels were observed in PBMC from HCV-infected naïve patients as compared to healthy donors. In healthy donors, IFNAR-1 mRNA levels were independent from IL-28B genotype, while in HCV patients, an increasing gradient was observed in TT vs CT vs CC carriers. In the latter group, a direct correlation between IFNAR-1 and endogenous IL-28B expression was observed. Moreover, IFN-lambda up-regulated IFNAR-1 expression in normal PBMC in a time-and dose-dependent manner, with a more effective response in CC vs TT carriers.

**Conclusion:**

Endogenous levels of IFN-lambda may be responsible for partial restoration of IFNAR-1 expression in HCV patients with favourable IL-28 genotype. This, in turn, may confer to CC carriers a response advantage to either endogenous or exogenous IFN-alpha, representing the biological basis for the observed association between CC genotype and favourable outcome of either natural infection (clearance vs chronicization) or IFN therapy.

## Introduction

Hepatitis C virus (HCV) is the leading cause of chronic liver disease, cirrhosis, and hepatocellular carcinoma in developed countries [1]. The ability of HCV to inhibit the activation of the endogenous type I interferon (IFN) system could underlie its success in establishing a chronic infection [2]. Type I IFNs are not only crucial factors in the innate immune system but are also the most important components of current therapy against chronic hepatitis C (CHC). In fact, the standard of care (SOC) for chronic HCV infection consists of pegylated IFN (peg-IFN)-2a or -2b and ribavirin (RBV). This treatment produces sustained virologic response (SVR) in only 40–50% of patients with HCV genotype 1 and approximately 60% in those infected with genotype 4, whereas over 80% of patients with genotype 2 or 3 achieve SVR [3], [4]. Moreover, several studies showed a correlation between pre-treatment expression levels of IFN stimulated genes (ISGs) and response to treatment, using either liver tissues or serum or peripheral blood mononuclear cells (PBMC) [5], [6]. Notably, patients with pre-elevated expression of ISGs (including MxA, PKR and ISG15) in the liver and/or PBMC showed a poor response to SOC as compared to patients with low basal levels [7], [8], [9].

A group of recently discovered cytokines (IFN-lambda1/interleukin-29 [IL-29], IFN-lambda2/IL-28A, and IFN-lambda3/IL-28B), assigned to a new type of IFN (type III IFN) gained increased attention in the HCV field [10].

Genome-wide association studies (GWAS) identified several single-nucleotide polymorphisms (SNPs) in IL-28B gene region, that were strongly related to therapy-induced HCV clearance rate in CHC patients [11], [12], [13]. Numerous investigators confirmed the importance of IL-28B SNPs in the treatment response [14], [15], [16]. Among the identified SNPs, rs12979860, located 3 kb upstream of the IL-28B gene, appeared as the most relevant, being the rs12979860-favorable CC genotype associated with a more than two-fold increased rate of SVR with respect to hapless (CT or TT) genotypes [17]. However, despite the existing clear evidence of association of IL-28B polymorphisms on spontaneous or therapy-induced resolution of the infection, the mechanisms triggered by these polymorphisms and their real biological consequences remain unexplained, and attract intensive investigation. A role for increased endogenous levels of IFN-lambda as the basis for these mechanisms is suggested by a number of studies. For instance, the expression of IL-28B was reported to be lower in whole blood or PBMC from individuals carrying the hapless genotypes as compared to those carrying the CC alleles [12], [13]. In another study by Langhans and others (2011), HCV chronically infected patients showed lower levels of circulating IFN-lambda as compared to spontaneously resolved infections and increased IFN-lambda levels were observed in carriers of the favorable CC rs12979860 [18]. Moreover, the association between increased IL-29 levels and CC allele has been reported in a recent study focused on therapy response [19].

TypeI and III IFNs exhibit a distinct receptor complex but common functions, both in term of broad range of antiviral mechanisms, induction of the same pattern of ISGs, and stimulation of immune effector functions. Despite the functional similarity with type I IFNs, clear evidence exists that type III IFNs have unique mechanisms of action, concerning, in particular, the kinetics of signal transduction after receptor engagement. Conflicting data exist on the relationship between genetic variation of IL-28 and the basal ISGs levels. Recent studies have suggested that baseline hepatic expression of ISGs was associated with genetic variation of IL-28B [20], [21], [22], while two studies reported that ISGs expression was associated with SVR, independently from IL-28B genotype [23], [24].

In a previous study we have shown that reduced expression of the IFN-alpha receptor -1 (IFNAR-1) may represent the biological basis for reduced response to SOC in poorly performing patients [25], such as HIV-coinfected individuals, hampering their ability to mount an appropriate response to exogenously administered IFN-alpha. However, so far no data are available on the correlation between IL-28 genotype and IFNAR-1 expression.

The aim of the present study was to establish whether the IL-28B rs12979860 genotype could influence the endogenous expression IFNAR-1, and to explore the possible role of IFN-lambda.

## Methods

### Ethics statement

The project was approved by Comitato Etico “Istituto Nazionale per le Malattie Infettive L. Spallanzani, I.R.C.C.S.” and patients agreed to participate to the study by signing informed consent.

### Patients

PBMC and plasma samples from 40 treatment-naive patients, chronically infected with HCV (genotype 1 or 4), collected at the National Institute for Infectious Diseases and stored in the Institutional Biorepository, for whom IL-28B genotype at locus rs12979860 was known, were retrospectively selected, in order to include a sufficient number of patients harboring the CC genotype (that is relatively rare in the Italian population) to perform a comparative analysis. The distribution of IL-28B genotypes was: 9 CC (22.5%), 18 CT (45%), 13 TT (32.5%). Clinical characteristics of patients are shown in Table 1. All patients were further characterized for IFN system activation in their PBMC. As control group, 6 healthy donors, matching the anagraphic characteristics of the HCV-positive group, were included (3 donors with IL-28B rs12979860 CC genotype, and 3 donors with TT genotype)

**Table 1:** Characteristics of 40 treatment naive HCV-infected patients included in the study.

**Age [median(range)] years**	53 (30–81)
**Sex (Male/Female)**	30/10
**AST [median(range)] IU/L**	45 (17–162)
**ALT [median(range)] IU/L**	62 (13–310)
**γ-GT [median(range)] IU/L**	40 (9–599)
**HCV load [median(range)] Log_10_ IU/ml**	6.12 (4.35–6.84)
**HCV genotypes (gt1/gt4)**	27/13
**rs 12979860 IL28B genotypes (CC/CT/TT)**	9/18/13

### IL-28B polymorphisms genotyping

IL-28B rs12979860 CC/CT/TT genotype was established on genomic DNA, using a custom made TaqMan assay with the following amplification primers: 5′ GCC TGT CGT GTA CTG AAC CA 3′ and 5′ GCG CGG AGT GCA ATT CAA C 3′, and TaqMan probes: VIC- TGG TTC GCG CCT TC-MGB and FAM-CTG GTT CAC GCC TTC–MGB. Polymerase chain reactions (PCR) were performed with an SDS 7900 ht qPCR thermocycler (Applied Biosystems, Forster City, CA) with the following amplification protocols: denaturation at 95°C for 10 minutes, followed by 40 cycles of denaturation at 92°C for 15 sec, and finished with annealing and extension at 60°C for a 1 minute. Genotype was attributed by SDS 1.3 software for allelic discrimination.

### mRNA levels of IFNAR-1 in PBMC of naive HCV-infected patients carrying different IL-28B rs12979860 genotypes before and after exposure to IFN-alpha

PBMC obtained by Ficoll/hyPaque (Pharmacia, Sweden) separation, frozen under liquid nitrogen, were thawed, suspended at 2×10^6^/mL in RPMI medium supplemented with 10% foetal bovine serum and then cultured for 3 h in the absence or presence of 10^3^ IU/ml human recombinant IFN-alpha2b (Intron; Schering Corp., Kenilworth, NJ, USA; specific activity: 400 MIU/mg,1 IU corresponding to 2.5 pg). Total cellular RNA was extracted from PBMC using Trizol (Gibco BRL, Grand Island, NY, USA) and reverse-transcribed by TaqMan Reverse Transcription Reagent kit (Applied Biosystems, Foster City, CA, USA) before and after treatment with IFN-alpha. The quantification of IFNAR-1 mRNA was performed by real-time RT-PCR; the results were normalized using beta-actin as housekeeping gene, and the results were expressed as ratio of IFNAR-1/beta-actin mRNA copy number, as previously described [26]. Basal levels of IFNAR-1 mRNA were measured in freshly thawed PBMC, without further incubation.

### IFN-lambda expression in HCV-infected patients

Basal mRNA levels for IFN-lambda were measured in freshly thawed PBMC by quantitative Real-time RT-PCR, according to a previously described method [27]. Standard curves were prepared with serial dilution of recombinant plasmid containing the target region, and the results were expressed as ratio to beta-actin.

Plasma levels of IFN-lambda protein were measured by enzyme-linked immunosorbent assay (ELISA) namely DuoSet human IFN-lambda 1/3, measuring all isotypes of Hu-IFN-lambda (IL-29, IL-28A, IL-28B), purchased from R&D System, Inc, Minneapolis, MN, USA. Result were expressed as pg/ml.

### mRNA levels of IFNAR-1 in PBMC of healthy donors before and after exposure to IFN-lambda

PBMC from healthy donors carrying different IL-28B genotypes were exposed either to 10 ng/ml or 100 ng/ml human recombinant IL-28/IFN-lambda2 (PBL, Interferon source, Piscatway, NJ, USA; ED50: 10–50 ng/mL) for different time points. Total cellular RNA was extracted by Trizol and IFNAR-1 mRNA levels were measured by real-time PCR as described above.

### Statistical analysis

Statistical analyses were performed by Prism 4 software (GraphPad, San Diego, CA). Differences were evaluated by the non parametric Mann-Whitney U test or by Student's t test, as appropriate. Correlations were analyzed by Pearson r test. Differences with p<0.05 were considered statistically significant.

## Results

### Levels of IFNAR-1 in PBMC from naive HCV-infected patients carrying different IL-28B rs12979860 genotypes, and in vitro response to IFN-alpha

**Figure 1:**
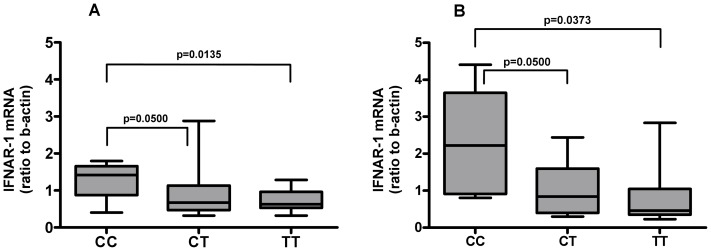
IFNAR-1 mRNA levels before and after treatment with IFN-alpha in PBMC from naive HCV-infected patients. Total cellular RNA was extracted and reverse-transcribed from PBMC of naive HCV-infected patients carrying different IL-28B rs12979869 genotypes CC, TT and CT before (**Panel A**) and after 3 h of exposure to 10^3^ IU/ml IFN-alpha (**Panel B**), then mRNA levels for IFNAR-1 were measured. Results are expressed as ratio to beta-actin (median, IQR).

To assess the relationships between the expression of IFNAR-1 mRNA and the IL-28B rs12979860 genotypes, mRNA levels of IFNAR-1 were tested at baseline and after 3 h of exposure to either control medium or IFN-alpha (10^3^ IU/ml) in PBMC obtained from naive HCV-infected patients carrying different IL-28B rs12979860 genotypes. Patients carrying CC genotype showed IFNAR-1 mRNA median basal levels significantly higher than patients with CT/TT genotype: 1.420 (IQR: 0.875–1.655) vs 0.629 (IQR: 0.504–1.005); p = 0.0142; in addition, significantly higher levels in CC vs CT/TT genotypes was observed after exposure to IFN-alpha [median: 2.220 (IQR: 0.908–3.647) vs 0.6280 (IQR: 0.395–1.522); p = 0.0149]. More in detail, the most prominent difference was observed between CC and TT groups, both at basal level (Fig. 1, Panel A) [median: 1.420 (IQR: 0.875–1.655) vs 0.629 (IQR: 0.532–0.925); p = 0.0135] and after exposure to IFN-alpha (Fig. 1, Panel B) [median: 2.220 (IQR: 0.908–3.647) vs 0.461 (IQR: 0.353–1.048); p = 0.0373], while between CC and CT groups a borderline significant difference was observed both at basal level (Fig. 1, Panel A) [median: 1.420 (IQR: 0.875–1.655) vs 0.676 (IQR: 0.468–1.137); p = 0.0500] and after exposure to IFN-alpha (Fig. 1, Panel B) [median values: 2.220 (IQR: 0.908–3.647) vs 0.840 (IQR: 0.397–1.599); p = 0.0500]. IFN-alpha treatment did not significantly affect the levels of IFNAR-1 mRNA in all genotypes (CC, untreated vs IFN-treated, median: 1.420 (IQR: 0.875–1.655) vs 2.034 (IQR: 0.808–2.888); p = 0.3510; CT, untreated vs IFN-treated CT, median: 0.6760 (IQR: 0.468–1.137) vs 1.046 (IQR: 0.411–1.639); p = 0.5510; TT, untreated vs IFN-treated, median: 0.6290 (IQR: 0.532–0.965) vs 0.461 (IQR: 0.353–1.048); p = 0.5240).

Basal levels of IFN-lambda was also investigated, and no differences were appreciated between the different IL-28B genotypes, at both PBMC mRNA and plasma protein level (data not shown). However, a significant correlation between mRNA levels of IFN-lambda and IFNAR-1 was observed in CC carriers (r = 0.6881, p = 0.0433).

### Role of IFN-lambda on IFNAR-1 mRNA expression in PBMC from healthy donors carrying different IL-28B rs12979860 genotypes

**Figure 2:**
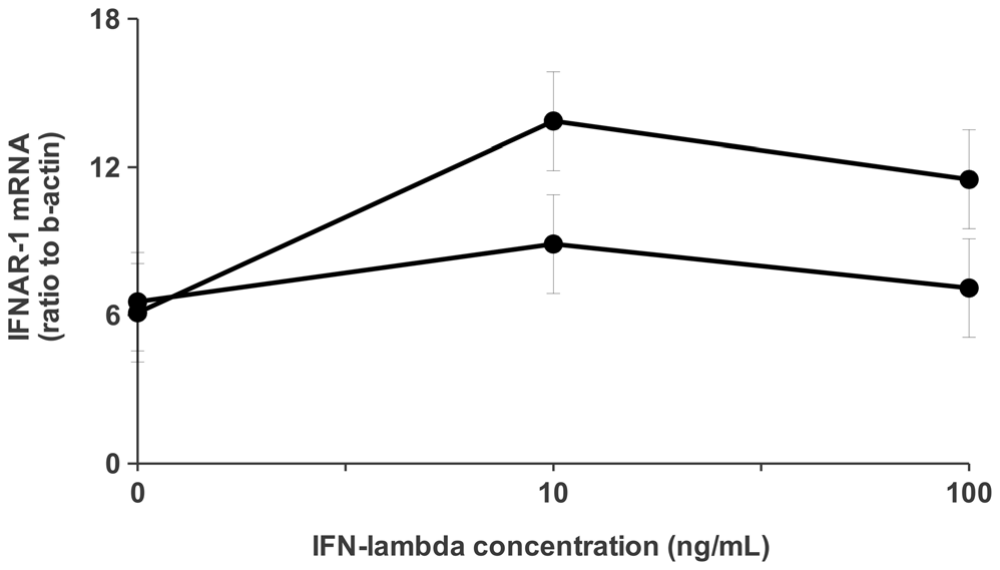
Dose dependent induction of IFNAR-1 mRNA levels following treatment with IFN-lambda in PBMC from healthy donors. PBMC from 6 healthy donors with different IL-28B rs12979860 genotypes (3 CC •; 3 TT ○) were exposed for 3 h to either control medium or IFN-lambda (10 ng/ml, 100 ng/ml), then mRNA levels for IFNAR-1 were measured. Results are expressed as ratio to beta-actin (mean ± SE).

To evaluate the possible role of IFN-lambda on IFNAR-1 mRNA expression, PBMC from 6 healthy donors carrying different IL-28B genotypes (3 rs12979860 CC, and 3 rs12979860 TT) were exposed either to 10 or 100 ng/ml of human recombinant IL-28/IFN-lambda2, and levels of IFNAR-1 mRNA were measured at different time points. The basal levels of IFNAR-1 mRNA in healthy donors were significantly higher than those observed in HCV patients [median: 6.516 (IQR: 4.888–7.556) vs 0.732 (IQR: 0.393–1.829); p = 0.0004], and seemed to be independent from IL-28B genotype. The dose dependent response after 3 h exposure to IFN-lambda is shown in Fig. 2, were up-regulation of IFNAR-1 mRNA was observed in both genotypes, being 10 ng/ml the most effective dose. More interestingly, the IFN-lambda-driven stimulation was more pronounced in subjects carrying the CC genotype at both doses.

**Figure 3:**
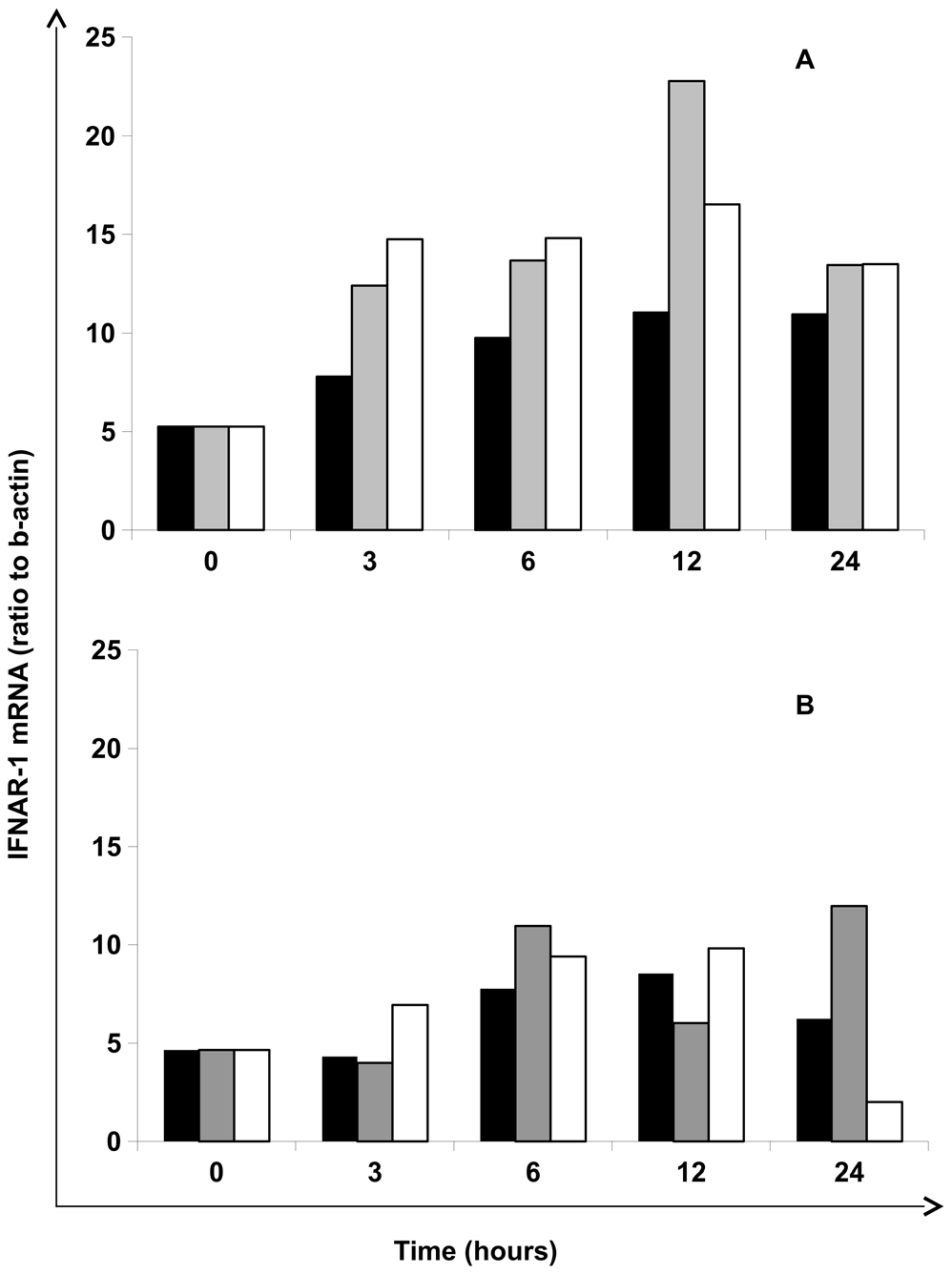
Time dependent induction of IFNAR-1 mRNA levels following treatment with IFN-lambda in PBMC from healthy donors. PBMC were exposed to control medium (▪) 10 ng/mL (▪) and 100 ng/mL (□) of IFN-lambda, then mRNA levels for IFNAR-1 were measured at different time points (0, 3, 6, 12 and 24 h). Results are expressed as ratio to beta-actin. Results from one representative experiment performed on PBMC from two healthy donors with IL-28B rs12979860 CC (**Panel A**) and TT (**Panel B**) genotype are shown.

In Fig. 3 time-and dose-dependent response from two representative subjects (CC and TT) is shown, confirming 10 ng/ml of IFN-lambda as optimal dose, and showing, again, a better stimulation in CC genotype. In particular, at 10 ng/ml peak stimulation in CC genotype occurred earlier (12 h) and was more extensive than in TT genotype (24 h).

## Discussion

Since 2009, several studies have shown that there is an important association between IL-28B polymorphisms and the outcome of standard anti-HCV therapy [9], [11]. Among the identified SNPs, rs12979860 appeared the most relevant, being associated with both therapy-induced and spontaneous clearance of HCV. Data so far available indicate that the rs12979860-favorable (CC) genotype is associated with higher expression of IFN-lambda [12], [13] that, in turn, may contribute to viral clearance. This hypothesis is further supported by the evidence that high IFN-lambda peak serum levels during the acute phase are associated with a self-limited course of HCV infection [18]. However, the association between elevated IFN-lambda expression and HCV clearance is not unequivocally reported, since other studies have shown higher levels of IFN-lambda in the liver of patients with the hapless genotype [28]. Inappropriate IFN-lambda activation (altered level, potency or timing) upon HCV infection may explain the apparent contradiction between the two opposite lines of evidence [29]. Based on these evidences and considering a previous study in which our group observed a reduced expression of IFNAR-1 mRNA in poorly performing patients [25]_,_ we explored the possible relationships between IL-28 rs12979860 genotype and the expression of IFNAR-1 in naive HCV patients.

Our results highlighted a significant difference of IFNAR-1 mRNA expression in PBMC from patients carrying rs12979860 CC and CT/TT genotypes, with the most prominent difference in the absence of -C allele. It is to be underlined that the IFN-alpha treatment did not significantly affect the levels of IFNAR-1 mRNA, suggesting a marginal role of this cytokine in the observed differences. A significant correlation between endogenous levels of circulating IFN-lambda and spontaneous levels of IFNAR-1 mRNA in PBMC was observed in patients carrying CC genotype, although no differences of IFN-lambda levels could be appreciated between the different genotypes. The lack of detectable difference is probably due to the chronic infection state of our patients, where the reduced levels of circulating IFN-lambda might have hampered the comparison. This is in line with other groups that described lower levels of circulating IFN-lambda in HCV chronically infected patients as compared to spontaneously resolved infections [18], [27]. We then explored whether IFN-lambda could be able to up-regulate IFNAR-1 expression in normal PBMC. As compared to HCV patients, the basal levels of IFNAR-1 mRNA in healthy donors appeared significantly higher, and seemed independent from IL-28 genotype. On the contrary, the IL-28 genotype was important in the IFN-lambda response, since a stimulation was observed in a time-and dose-dependent manner, and was much more pronounced in CC vs TT carriers.

Our findings suggest that IFN-lambda could play a crucial role in the modulation of IFNAR-1 expression, and that endogenous levels of IL-28B may be responsible for partial restoration of IFNAR-1 expression in HCV patients with favourable IL-28B genotypes. A number of published studies, show that the mRNA levels of IFNAR-1 are correlated with the extent of IFN response both in vivo and in vitro [30], [31], [32]. Therefore, although the present study does not provide direct demonstration that the different IFNAR-1 mRNA levels translate into different expression of IFNAR at cell surface, it is tempting to speculate that this partial restoration may confer to CC carriers a response advantage to either endogenous or exogenous IFN-alpha, representing the biological basis for the observed association between CC genotype and favourable outcome of either natural infection (clearance vs chronicization) or IFN therapy.

In summary, although the findings from the present study are preliminary, since they derive from a limited number of patients and might benefit from larger studies, they provide novel information, contributing to elucidate the mechanisms underlying the strong predictive value of IL-28B polymorphisms on the natural history and on the response to IFN therapy in HCV infection.
